# Toward a New Kind of Vaccine: A Logical Extension of the Symmetrical Immune Network Theory

**DOI:** 10.2196/ijmr.7612

**Published:** 2017-07-05

**Authors:** Reginald Gorczynski, Geoffrey Hoffmann

**Affiliations:** ^1^ University of Toronto Toronto, ON Canada; ^2^ UBC Vancouver, BC Canada

**Keywords:** immunity, anti-idiotype antibodies immune system concepts, information networks

## Abstract

**Background:**

The symmetrical immune network theory, developed in 1975, is based on the existence of specific T cell factors and hypothesizes that normal IgG immune responses comprise the production of 2 kinds of antibodies, namely antigen-specific antibodies and anti-idiotypic antibodies.

**Objective:**

The aim of this study was to confirm the existence of specific T cells factors and to show that immunization of C3H mice with BL/6 skin or using nominal antigen for immunization (Tetanus Toxoid) induced production of antigen-specific (anti-BL/6 or antitetanus) antibodies plus anti-idiotypic antibodies (C3H anti-anti-C3H). Subsequently, we investigated the role of combinations of antigen-specific and anti-idiotype antibodies in a variety of animal models of clinical diseases.

**Methods:**

Antigen-specific antibodies were produced by conventional immunization of mice (eg, with tetanus toxoid or by skin allografting). Subsequent anti-idiotypic antibodies were derived by exhaustive absorption of antigen-specific antibody, with confirmation of anti-idiotypic specificity by binding to relevant target antigen-specific antibodies in an enzyme-linked immunosorbent assay (ELISA). Antigen-specific plus anti-idiotypic antibodies were then used to modulate skin allograft survival, dextran sulfate sodium (DSS)-induced colitis, *ovalbumin* (OVA)-induced IgE production, and breast cancer growth in mice.

**Results:**

Infusions of anti-BL/6 antibodies together with BL/6 anti-anti-BL/6 antibodies specifically suppressed (>85%) an immune response to BL/6 lymphocytes in C3H mice. The two kinds of antibodies with complementary specificity are hypothesized to stimulate 2 populations of T lymphocytes. Coselection of these 2 populations leads to a new stable steady state of the system with diminished reactivity to BL/6 tissue. A combination of anti-C3H and C3H anti‑anti-C3H IgG antibodies down-regulated inflammation in a mouse model of inflammatory bowel disease (>75%) and attenuated anti-IgE production and sensitization to produce IL4 cytokines (>70%) in an OVA-allergy model. Combination of C3H anti‑BL/6 and BL/6 anti-anti-BL/6 antibodies decreased tumor growth and metastases (>705) in an EMT6 transplantable breast cancer model.

**Conclusions:**

Use of a combination of antigen-specific and anti-idiotypic antibodies has potential as a new class of vaccines.

## Introduction

Much research on anti-idiotypic antibodies has been focused on such antibodies mimicking the shape of the antigen, and therefore, being able to substitute for the antigen [1-3]. An antibody specific for an antigen X has a V region that is anti-X, which we refer to as an anti-X idiotype. If we immunize a rabbit with an anti-X antibody, together with an adjuvant, the rabbit may make anti-anti-X anti-idiotypic antibodies. However, such anti-idiotypic antibodies play no role in the immune network theory that has been developed in a series of papers and a monograph published from 1975 till date [4-7]. The theory is called the symmetrical immune network theory. The anti-idiotypes that play a role in immune network theory are of two types, namely coselection anti-idiotypes [5] and second symmetry anti-idiotypes [4]. Coselection anti-idiotypes are expressed by lymphocytes that are coselected (mutually selected) with antigen-specific lymphocytes, whereas second symmetry anti-idiotypes are anti-idiotypes present in an A anti-B or A anti-X serum that are specific for antigen-specific antibodies present in a B anti-A serum and where X is again an antigen.

**Figure 1:**
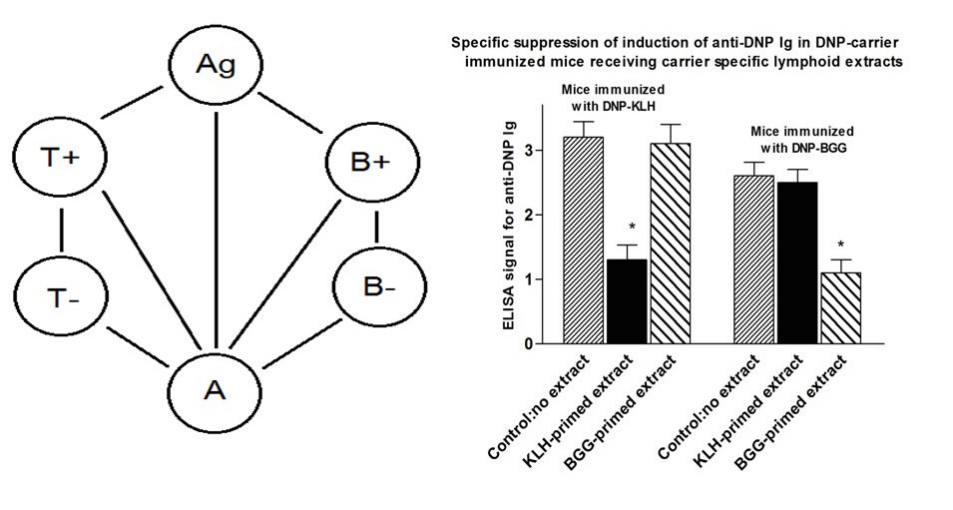
A simple idiotypic network model that includes antigen (Ag), antigen-specific T cells and B cells (T+ and B+), anti-idiotypic T cells and B cells (T- and B-) and non-specific accessory cells (A cells; see left hand panel). For explanation, see the sections in the text on the role of specific T cell factors in an immune response and the role of specific T cell factors in the induction of tolerance by an antigen.Repeat of Takemori and Tada study (right hand panel-see text for details). *P<.05 compared with no extract control (MANOVA).

The symmetrical immune network theory involves symmetrical stimulatory, inhibitory, and killing interactions. A simple symmetrical network model that includes antigen (Ag), antigen‑specific lymphocytes (T+ and B+), anti-idiotypic lymphocytes (T− and B−), and nonspecific accessory (A) cells is shown in Figure 1. A cells include monocytes, macrophages, and dendritic cells. This model will be described below in the sections on the roles of specific T cell factors. In this model, an immune response involves an immunogenic form of an antigen stimulating T and B cells, specific T cell factors from the T cells binding to a receptor on the surface of A cells, and the antigen activating the A cell by cross-linking a receptor on the A cell via the specific T cell factors. The antigen also stimulates antigen-specific B cells by cross-linking their receptors, and they proliferate and express a receptor for the differentiation factor produced by the activated A cells. When the antigen‑specific B cells receive the second signal differentiation factor, they switch to being antibody-secreting plasma cells.

Controversially, the symmetrical immune network theory involves a role for antigen-specific T cell factors. Many papers published in the 1970s and 1980s demonstrated the existence of such factors [8-18], including a paper by Takemori and Tada that rigorously demonstrated that carrier-specific T cell factors can specifically suppress IgG responses [10]. In 1991, GWH pointed out that the reproducibility of the Takemori-Tada result was a suitable test for the existence of these factors [18]. If that experiment could not be reproduced, it would call into question the fundamental ideas contained within the symmetrical immune network theory. On the other hand, if the experiment was reproduced, such factors presumably do indeed exist and their existence is thus currently erroneously being widely ignored. To our knowledge, in the 25 years since 1991, no one has claimed to be unable to reproduce the Takemori-Tada result, nor has anyone published confirmation that the experiment is reproducible. As a starting point for our further analysis of the importance of a symmetrical immune network theory to offer new insight into how to control various immune reactivities of clinical significance, we first attempted to confirm the reproducibility of this key experiment (see Figure 1).

In the studies described below, we have moved beyond this important confirmation of the existence of T cell derived antigen-specific suppressive factors to provide evidence that application of symmetrical immune network theory provides an invaluable means to alter, in separate mouse model systems, transplant rejection, inflammatory bowel disease, allergic responses, and tumor growth.

**Figure 2:**
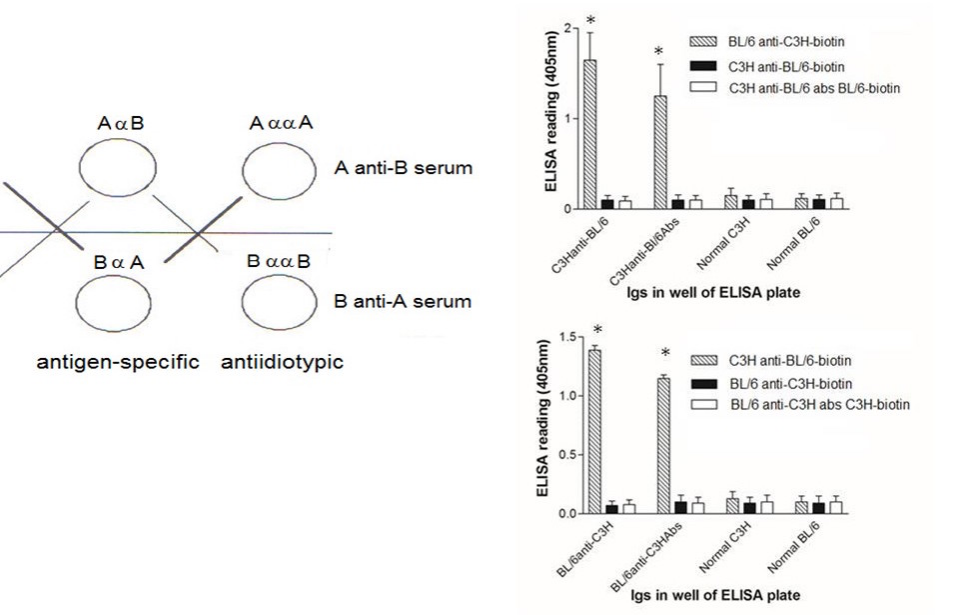
Left hand panel shows the antibodies in an A anti-B serum, where A and B are two strains of mice, including A anti-B and A anti-anti-A antibodies (shown as AαB and AααA, respectively). These are complementary to the B anti-A and B anti-anti-B antibodies that are present in a B anti-A serum.Right hand panel shows binding of complementary antibodies assayed by ELISA. Biotinylated BL/6 anti-C3H IgG binds to C3H anti-BL/6 IgG and to C3H anti-BL/6 IgG absorbed with BL/6 lymphocytes (upper panel), while C3H anti-BL/6 IgG binds to BL/6 anti-C3H IgG and BL/6 anti-C3H IgG absorbed with C3H lymphocytes (lower panel). ELISA plates were coated with10ng IgG. Biotinylated IgGs were used at 1:2500 concentration (with streptavidin-HRP at 1:2000). *P<.02 MANOVA.

## Methods

### Mice

Female C57BL/6, C3H/HEJ and BALB/c mice were purchased from Jax Labs, Bar Harbour Maine. All mice were housed 5 per cage and maintained at the University Health Network (UHN) animal facility (animal protocol: RMG: AUP1.18). Mice were used at 8 weeks of age.

### Reproduction of Takemori and Tada Study

To prepare T cell extracts, 5 BALB/c mice per group were immunized x2 with 100µg/mouse keyhole limpet hemocyanin (KLH) or *bovine gamma globulin* (BGG) (both purchased from Sigma Biochemicals, Canada) at 14d intervals. Mice were sacrificed 14d after the last immunization, spleen+ thymus cells pooled within groups, and cells resuspended at 3x10^8^/ml. Suspensions were sonicated at 4°C for 4 min and subjected to ultracentrifugation for 60 min at 4°C. Additionally, 0.3ml of sonicate per mouse was infused into groups of 10 BALB/c. However, 10 control mice received no extract.

A total of 5 mice of each group of 10 mice were subsequently immunized with either dinitrophenol (DNP)-KLH of DNP-BGG (100µg/mouse) in complete Freund’s adjuvant (Sigma Biochemicals, Canada). Mice were sacrificed 12d later and serum collected from all individuals. Sera were assayed in ELISA plates that were precoated with DNP-coupled albumin (100ng/well) and with horseradish *peroxidase* (HRP)-anti-mouse Ig (Cedarlane Labs, Burlington, Canada) as developing Ig. Test sera were tested at 1:5, 1:20, and 1:100 dilution-only; data for 1:20 dilutions are shown in the Results section (see Figure 1, pooled from 2 studies).

### Skin Grafting

C3H or BL/6 mice received allogeneic or syngeneic skin grafts transplanted to the flank as described previously [19]. Graft survival was monitored daily by an observer blinded to any previous treatment of the graft recipients.

**Figure 3:**
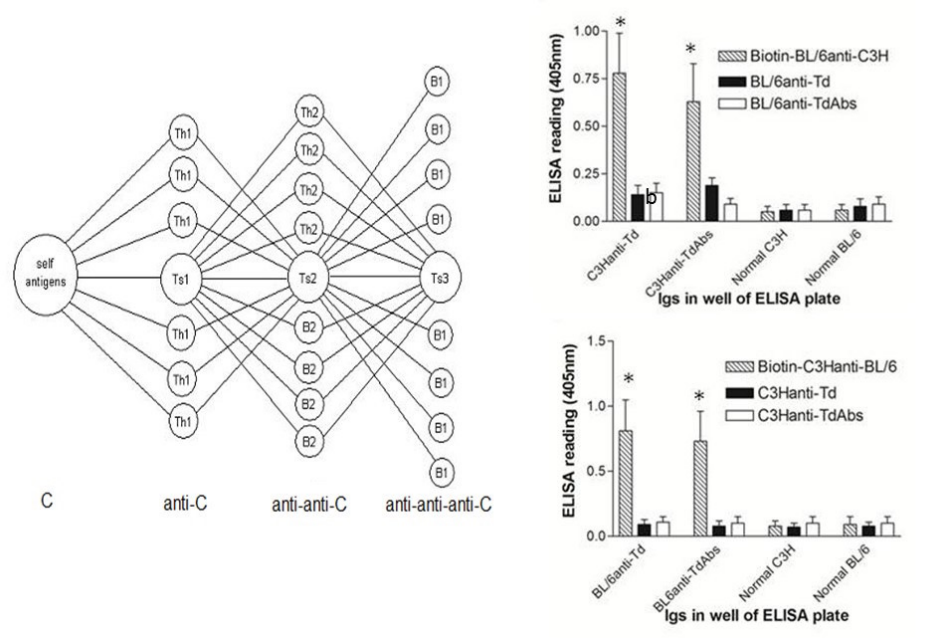
In the symmetrical immune network theory (left hand panel), self antigens of a vertebrate C stimulate Th1 and Ts1 lymphocytes (anti-C), which are co-selected with Th2, Ts2 and B2 lymphocytes (anti-anti-C), which in turn co-select Ts3 and B1 lymphocytes (anti-anti-anti-C). B2 cells are IgG-secreting B lymphocytes, and B1 cells are IgM‑secreting B lymphocytes. Anti-foreign and anti‑anti-self antibodies are produced in BL/6 and C3H mice immunized with tetanus toxoid (Td)-right hand panels. All IgGs were coated on ELISA plates at 50ng/well. All developing sera were used at 1:400 concentration, with streptavidin HRP used at 1:2000. In the upper panel, biotinylated BL/6 anti-C3H IgG binds to C3H anti-Td IgG and to C3H anti-Td IgG absorbed with Td. The lower panel shows the converse, with biotinylated C3H anti-BL/6 IgG binding to BL/6 anti-Td IgG. All data are from a total of 10 mice/group; *P<.05, MANOVA.

In some cases, grafted mice were used as sources of antigraft specific Ig (or anti-anti-self Ig). In these instances, 15 mice per group were grafted twice (at 21d intervals), with donor skin (same donor haplotype) and blood obtained by cardiac puncture 10d after the second graft. Pooled serum was obtained by centrifugation (5000g at 4°C for 20 min), heat inactivated, aliquoted (0.3ml aliquots), and stored at −80°C. Where serum was absorbed (anti-anti-donor Ig), aliquots (0.3ml) were absorbed 3x for 60 min at room temperature with a fresh pellet of 3x10^8^ spleen and thymus, prepared from the described donors (20 mice/group used as donors for absorption). Following 3 serial absorptions, depletion of cytotoxic activity in serum was confirmed using spleen cell blasts and serial dilutions of antibody with rabbit complement (1:10 final dilution), incubated at 37°C for 60 min before addition of trypan blue to assess cell death. Routinely titres dropped from 50% lysis at ~1:2000 to ~1:2 following this absorption (Figures 2 and 3).

In other studies, mice were pretreated before transplantation with A anti-B sera and anti-anti-self Ig. In these cases, 8 mice per group received weekly injections (intravenous [IV] and/or intraperitoneal [IP]) of serum Ig at the concentration described, diluted in 0.3ml Phosphate buffered saline (PBS). Control serum in these studies (referred to as “normal IgG” in the text and Figures) represented serum pooled from a minimum of 15 equivalent nonimmunized naive mice.

### Mixed Leukocyte Culture Assays (MLCs) and Chromium-51 Lysis Assays for Cytotoxic T Lymphocytes (CTL)

**Figure 4:**
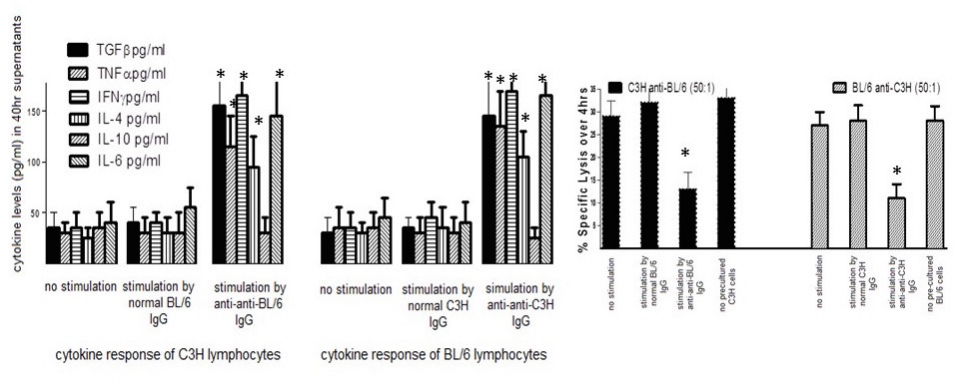
The 2 left hand panels show cytokine levels (measured in ELISA)from cultures of C3H splenocytes with no stimulation or stimulated with 20μg/ml of normal BL/6 IgG or BL/6 anti-anti-BL/6 IgG-far left- or cytokines from BL/6 splenocytes with no stimulation, or stimulated by normal C3H IgG or C3H anti-anti-C3H IgG. *P<.05 compared with corresponding no stimulation controls (MANOVA)The right hand panel shows antigen-specific Tregs in cultures of C3H (left) or BL/6 (right) splenocytes induced by anti-idiotypic antibodies from mice with cytokine production shown. Control cells received stimulation with 20μg/ml of either normal BL/6 IgG or no stimulation. Tregs were harvested at 96hr and added to mixtures of C3H (or BL/6) cells stimulated with irradiated BL/6 (or C3H) cells. CTL were assayed at 5d in 51Cr release assays. *P<.05 compared with corresponding controls not receiving Tregs (far right in each panel) by MANOVA.

**Figure 5:**
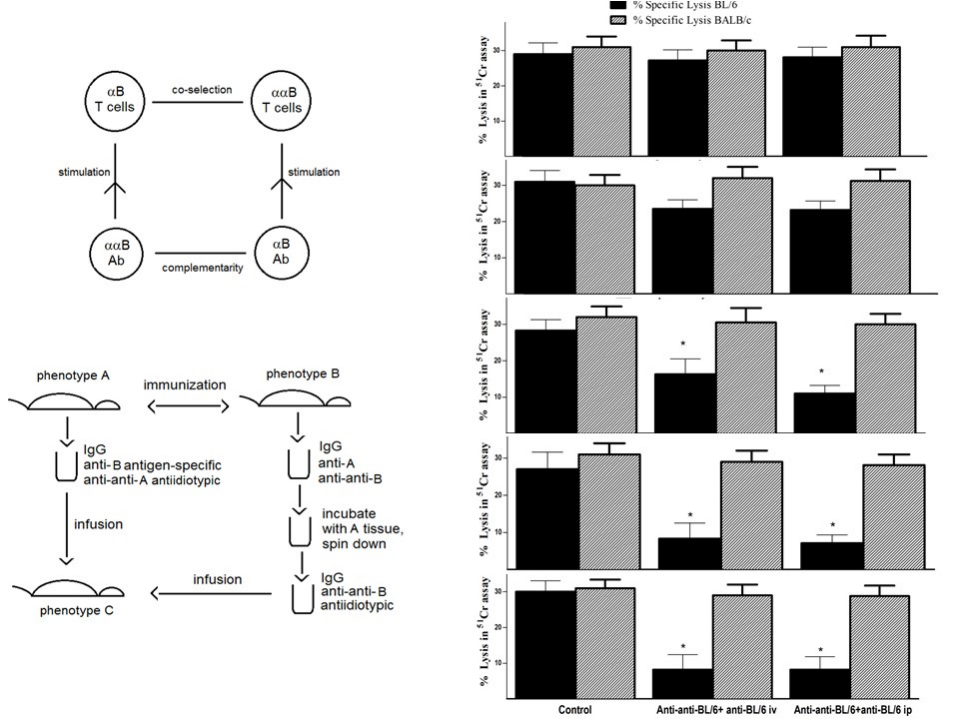
The left hand upper panel shows a co-selection mechanism for induction of transplantation tolerance in vertebrate C based on the interactions of Figure 2. Anti-B antibodies stimulate anti-anti-BT cells and the anti-anti-B antibodies stimulate anti-B T cells. There is co-selection of the anti-anti-B T cells and the anti-B T cells, taking the immune system of C to a state in which there are elevated levels of these two T cell populations. This is a state of the vertebrate C that is specifically suppressed with respect to making an immune response to B.The lower panel shows a polyclonal version of this vaccination. Immunization of strain A with strain B tissue causes production of anti-B plus anti‑anti-A IgG, while the converse immunization produces anti-A plus anti-anti-B IgG. The anti-A antibodies in the latter IgG are removed by absorption with A tissue (lymphocytes) yielding anti‑anti-B IgG without anti-A antibodies. A vertebrate of phenotype C receives infusions of anti-anti-B plus anti-B IgG. C also receives anti-anti-A IgG, but in the absence of anti-A IgG there is no A-specific positive feedback loop.The right hand panels show the anti-BL/6 CTL response following treatment of 20C3H mice with weekly infusions of 1μg anti-BL/6 IgG plus 1μg anti-anti-BL/6 IgG, given either IV or IP. Sequential panels show the CTL responses after 2, 3, 4, 5 or 6 infusions respectively. CTL were measured in individual spleen samples after stimulation with BL/6 or third-party control (BALB/c) cells. Data show % lysis (20:1 effector target) in groups of 4 mice. * *P*<.05, MANOVA.

Graft recipients were sacrificed at the times described in the text and figure legends, and single cell spleen suspensions prepared for individual mice. 6x10^6^ responder cells were incubated in triplicate with 3x10^6^ irradiated spleen stimulator cells in flat bottomed culture wells (24-well culture plates) in 3.0 ml αMinimal Essential Medium (αMEM: Gibco, USA), supplemented with 10% of fetal calf serum and 10^-6^M 2-mercaptoethanol (αF10). In some cultures, an aliquot (200µl) of medium was removed at 40 h to assess cytokine production, using commercial ELISA kits (BioLegend, USA). After 5d, cells were harvested, washed, and used at different effector:target ratios (from 50:1 to 5:1) in triplicate for lysis over 5 h of 5x10^351^Cr-labeled 72 h ConA spleen cell blasts homologous with the cells used as stimulator in MLCs. Data shown in Figures 4 and 5 represent mean lysis at a 20:1 E:T ratio.

### Inflammatory Bowel Disease after Intravenous (IV) Ig Treatment

C57BL/6 female mice, with high susceptibility to dextran sulfate sodium (DSS)-induced colitis [20], purchased from the Jackson laboratories (Bar Harbor, ME) were used throughout. All mice were housed five per cage under specific pathogen-free conditions, allowed standard diet (or high fat) and water ad libitum, and used at 6-8 weeks of age. Where shown, mice received 10µg BL/6 anti-C3H (IP) and 10µg C3H anti-BL/6, absorbed with BL/6 (C3H anti-anti-C3H) IV weekly, beginning 14d before the first DSS treatment.

Animal experimentation was performed following guidelines of an accredited animal care committee (protocol no. AUP.1.18). Humane endpoints were used in all studies, with mice monitored daily. Animals were euthanized (overdose with pentobarbital) when they were exhibiting signs of distress (weight loss≥25%, hunched posture, diarrhea, and loss of active movements)—no mortality was seen in this chronic *inflammatory bowel disease* (IBD) protocol. Animals with diarrhea (but weight loss<25%) received daily IP injections of saline (1ml x3 at 8 h intervals) to avoid dehydration.

### Induction of Colitis

**Figure 6:**
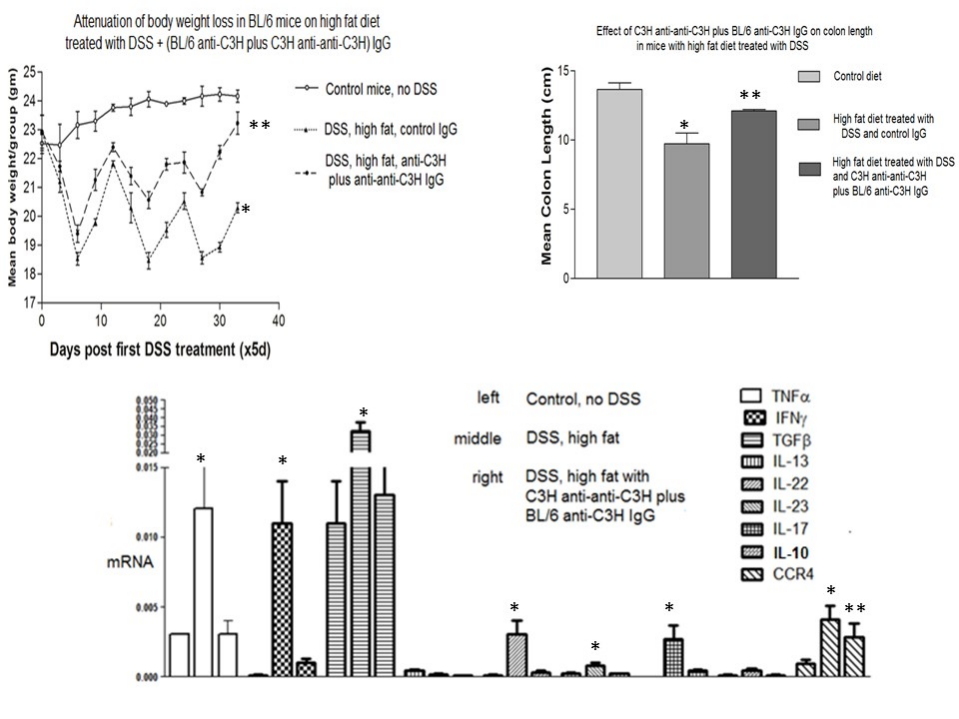
Inflammatory colitis in mice receiving C3H anti-anti-C3H antibodies plus BL/6 anti-C3H IgG antibodies. The upper left panel shows attenuation of weight loss in C57BL/6mice receiving antibody treatment. * *P*<.05 compared with control diet; ** *P*<.05 compared with high fat diet and control Ig (Mann-Whitney U test). The upper right panel shows diminished changes in colon length caused by DSS and high fat diet in mice receiving antibody treatment. * *P*<.05 compared with control diet; ** *P*<.05 compared with DSS/high fat diet and control Ig (Man Whitney U test). Finally, data in the lower panel shows attenuation of cytokine mRNA expression in DSS colitis after antibody infusion. Mice received 10µg of each of the antibodies intravenously on days -14 and -7 prior to the commencement of DSS and high fat diet on day 0. * *P*<.05 compared with control diet; ** *P*<.05 compared with high fat diet and control Ig (Mann-Whitney U test).

Chronic DSS colitis was induced by giving mice (5/group) distilled drinking water containing 3% (wt/vol) DSS (m.w.=40 kDa; ICN Biochemicals, Aurora, OH) for 5 d, followed by 7 d of normal drinking water for a total of 3 cycles. Controls received normal drinking water throughout the study. Body weight was measured 3 times a week throughout the experiment. No significant mortality was seen in any groups in the chronic colitis model. As shown in relevant Figure 6, the maximum weight loss observed with animals weighed daily was ~25%, with all mice recovering weight loss within 7-10 d post cessation of DSS exposure. All studies consisted of 5 mice/group receiving either normal water or DSS to drink. Groups treated with normal water or DSS also received treatment with control Ig or a combination of anti-anti-C3H and anti-C3H IgG antibodies as shown.

### Ribonucleic Acid (RNA) Isolation and Real-Time Reverse Transcription Polymerase Chain Reaction (RT-PCR)

Total ribonucleic acid (RNA) was isolated from colonic tissue using TRIzol reagent (Invitrogen Canada, Burlington, ON). Total RNA (1 μg) was treated with DNase I and then reverse transcribed using High Capacity cDNA Reverse Transcription Kit (Applied Biosystems, Foster city, CA) following the manufacturer’s instruction. First strand complementary deoxyribonucleic acid (cDNA) was diluted 1:20 and used for quantitative polymerase chain reaction (PCR) on an ABI 7900HT sequence detection system with SYBR green master mix (Applied Biosystems). Messenger RNA levels were normalized to a composite of glyceraldehyde 3-phosphate dehydrogenase (GAPDH) and hypoxanthine-guanine phosphoribosyltransferase (HPRT) expression levels. Experiments were repeated 3 times with 3 independent cDNA syntheses. The primers used for real-time reverse transcription polymerase chain reaction (RT-PCR) are shown in Table 1 below.

**Table 1:** Primers used for real-time PCR.

Cytokine or chemokine	Forward	Reverse
IFN-γ	5'-TATTGCCAAGTTTGAGGTCAACA-3ʹ	5'-GCTGGATTCCGGCAACAG-3'
TNF-α	5'-AGACCCTCACACTCAGATCATCTTC-3’	5’-CCACTTGGTGGTTTGCTACGA- 3’
IL-1β	5’-TCGTGCTGTCGGACCCATAT-3’	5’-GGTTCTCCTTGTACAAAGCTCATG-3’
IL-4	5’-TCATCGGCATTTTGAACGAG-3’	5’-TTTGGCACATCCATCTCCG-3’
IL-6	5’-CTCTGGGAAATCGTGGAAATG-3’	5’-CAGATTGTTTTCTGCAAGTGCAT-3’
IL-10	5’-AAGGCAGTGGAGCAGGTGAA-3’	5’-TTCTATGCAGTTGATGAAGATGTCAA-3’
IL-12	5’-CCCAAGGTCAGCGTTCCA-3’	5’-GGCAAGGGTGGCCAAAA-3’
IL-17	5’-CTCAGACTACCTCAACCGTTCCA-3’	5’-CCAGATCACAGAGGGATATCTATCAG-3’
IL-22	5’-GTGCCTTTCCTGACCAAA-3’	5’-TCTCCTTCAGCCTTCTGA-3’
IL-23	5’-GACAACAGCCAGTTCTGCTT-3’	5’-AGGGAGGTGTGAAGTTGCTC-3’
IL-23R	5’-AATTTGACGCCAATTTCACA-3’	5’-ACCAGTTTCTTGACATCGCA-3’
TGF-β	5’-CGAAGCGGACTACTATGCTAAAGA-3’	5’-GTTTTCTCATAGATGGCGTTGTTG-3’
Foxp3	5’-AGTCTGCAAGTGGCCTGGTT-3’	5’-GGGCCTTGCCTTTCTCATC-3’
CCR4	5’-AGACTGTCCTCAGGATCACTTTCA-3’	5’-CCGGGTACCAGCAGGAGAA-3’
CCL-17	5’-ATGCCATCGTGTTTCTGACTGT-3’	5’-GCCTTGGGTTTTTCACCAATC-3’
CCL-22	5’-AAGCCTGGCGTTGTTTTGAT-3’	5’-AAGCCGAGTTCAGCAAAGTT-3’

### Induction of IgE after Ovalbumin (OVA) Immunization and Attenuation by IVIg

The protocol used to immunize BALB/c mice to produce IgE against OVA was essentially that described elsewhere [21]. A total of 8 mice per group received 10µg OVA emulsified in alum at day 0 and day 10. A control group received no OVA immunization. Beginning on day 7, groups of mice received normal saline IV, BL/6 anti-C3H Ig (10µg) IP weekly for 5 doses, C3H anti-anti-C3H Ig (10µg) IV weekly for 5 doses, or a combination of these latter 2 treatments. All 4 groups and the no OVA control received ongoing exposure to egg white solution in the drinking water until 42 d. At this time, all mice received a booster injection of OVA in alum, with sacrifice 7 d later.

**Figure 7:**
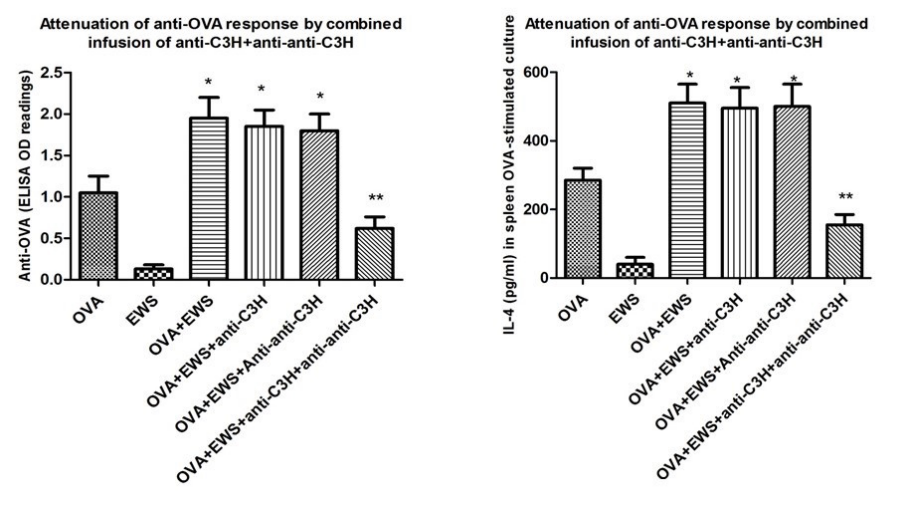
Attenuation of immune responses in 8/group OVA-immunized mice receiving combination treatment with anti-C3H and anti-anti-C3H Ig. Mice received OVA-in alum on days 0, 10, and 42, with some groups also receiving egg white solution (EWS) in the drinking water from days 14-42. Ig was infused weekly iv from day 7-35. Mice were sacrificed at day 49. Left hand panel shows IgE serum responses, while right hand panels shows attenuation of IL-4 production (ELISA) at 72hr from OVA-stimulated splenocytes of the same mice. *indicates significantly greater than controls receiving only OVA-in alum (far left), P<.05 (MANOVA); ** indicates significantly reduced relative to all other groups receiving OVA-alum and EWS, P<.05 (MANOVA).

On sacrifice, serum IgE to OVA was measured by ELISA using plates coated with 100ng/well of OVA and developed with HRP-anti-mouse IgE and appropriate substrate (Figure 7).

In addition, 5x10^6^ splenocytes from individual animals were challenged in vitro in 2ml medium, with 100µg/ml OVA for 72 h and IL-4 in culture supernatants assayed by ELISA (Figure 7).

### EMT6 Breast Cancer Model

**Figure 8:**
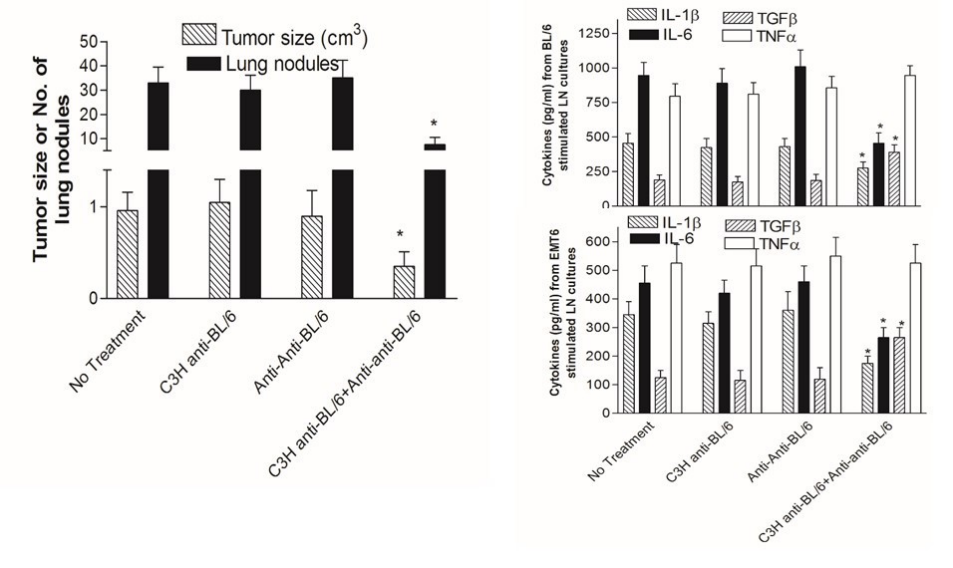
Decrease in tumor growth and metastases in BALB/c mice receiving anti-BL/6 and/or anti-anti-BL/6 Ig 14 and 7d before the transplantable breast cancer EMT6 (left hand panel). Injections of antibody were continued at 7d intervals until sacrifice. 14d after EMT6 injection tumors were resected and mice returned to their cages. Lung tumor nodules were measured 14d later. *P<.05 (MANOVA) compared with all other groups.The right hand panels show cytokine production (ELISA) from tumor-draining lymph nodes stimulated with either irradiated BL/6 splenocytes (upper panel) or EMT6 tumor cells (lower panel). *indicates P<.05 (MANOVA) compared with all other groups.

6 BALB/c mice per group received 5x10^5^ EMT6 tumor cells injected into the mammary fat pad, as described previously [22]. In experimental groups, the animals received weekly pretreatment with control Ig or a combination of anti-anti-C3H and anti-C3H IgG antibodies, beginning 14d before tumor injection. Tumor growth was monitored daily (Figure 8).

### Statistics

All data from experiments reported below are summed over at least two studies, with a minimum of 10 mice or combined groups for all experiments. In general, for studies with multiple groups, a multivariate analysis of variance (MANOVA) test was first applied to assess for any significant differences between groups, and subsequently, where indicated, paired *t*-tests were used to compare individual groups with the documented control. In some instances (noted in the Figure legends), a Mann-Whitney non parametric tests was used to compare groups.

## Results

### Confirmation of Evidence for Specific T Cell-Derived Suppressive Factors

As shown in Figure 1, in an experimental design closely following the original Takemori and Tada design (see Methods), BALB/c mice received a cell free extract derived from sonicated (spleen + thymocyte) cell preparations of mice primed twice on days 0 and 14 with 100µg of BGG or KLH. The extract was infused into 5 mice per group that subsequently received DNP coupled to BGG or DNP coupled to KLH. IgG responses to DNP were measured by ELISA.

The data show clearly that mice receiving KLH derived cell extract showed a suppressed response to DNP-KLH but not DNP-BGG and vice versa, implying, as initially suggested by Takemori and Tada, the existence of antigen-specific (T) lymphocyte derived factors [10,11]. As per the original hypothesis from Takemori and Tada, specific tolerance induction results from (1) a non-immunogenic form of an antigen stimulating T+ cells, (2) specific T cell factors from T+ cells binding to the surface of the A cells and stimulating T− cells, and (3) the T− cells secreting specific factors and stimulating the proliferation of T+ cells. The positive feedback loop involving antigen-specific T (T+) and anti-idiotypic (T−) cells takes the system to a suppressed stable steady state with elevated levels of the T+ and T− cell populations. There is coselection (mutual selection) of the idiotypic T cells and the anti-idiotypic T cells. The specific T cell factors have a molecular weight of about 50,000 Daltons and are therefore believed to be monovalent, in contrast to an IgG antibody that, with a molecular weight of 150,000, is divalent. The elevated levels of T+ and T− populations with their specific monovalent factors inhibit the stimulation of B+ and B− cells by the cross-linking of the B cell receptors. An immune response involves the antigen cross-linking a receptor on the A cell via adsorbed T+ factors, the A cell secreting a nonspecific differentiation factor, B cells being stimulated by antigen and expressing a receptor for the nonspecific factor, and on receipt of the differentiation factor switching from proliferation to antibody-secreting cells.

### Production and Testing of Anti-Idiotypic Antibodies

We showed previously that allo-immunization with lymphocytes could induce the production of anti-idiotypic antibodies, and small doses of these antibodies could subsequently enhance survival of allogeneic skin grafts [4]. The anti-idiotypic antibodies were produced by the strain of mice that was the donor of the skin grafts and were donor anti-anti-donor antibodies. Figure 2 shows the relationships of the antibodies in an A anti-B serum to the antibodies in a B anti-A serum according to the symmetrical immune network theory [4,23]. The antigen-specific antibodies include anti‑MHC class I antibodies and anti-I-J antibodies. The antigen-specific antibodies in an A anti-B serum are also specific for the anti-idiotypic antibodies (B anti-anti-B) in a B anti-A serum. Similarly, the antigen-specific antibodies in a B anti-A serum are specific for the anti-idiotypic antibodies (A anti-anti-A) in an A anti-B serum. This set of relationships is known as “second symmetry” [6,24]. (“First symmetry” in the symmetrical immune network theory is the concept that if an idiotype A is anti-idiotypic to an idiotype B, the idiotype B is anti-idiotypic to the idiotype A; reviewed in [6]).

In our previous work on second symmetry in mice, we generated A anti-anti-A antibodies (anti-idiotypic antibodies) by immunizing mice of strain A with strain B lymphocytes. The A anti-anti-A antibodies were assumed to be produced in response to the anti-A receptors of the strain B lymphocytes. We have extended these studies to show that there is no need to immunize strain A with lymphocytes per se to generate the A anti‑anti-A immune response. Rather, 2 rounds of B strain skin grafts on A strain mice also induce the production of A anti-anti-A antibodies in the A strain mice. This is shown explicitly in the data of ELISA assays in Figure 2

In these ELISA assays, normal IgG, alloimmune IgG, or IgG from alloimmune mice that had been absorbed using lymphocytes of the strain used in the skin grafting, were coated on ELISA plates at the dilutions shown in the caption of Figure 2. In all cases IgG was pooled from a total of 15 mice of each group. After incubation with biotinylated allo-antiserum, the bound antibodies were detected with streptavidin-alkaline phosphatase and substrate. The resulting signal is interpreted as resulting from the binding of biotinylated antigen-specific antibodies to complementary anti-idiotypic antibodies on the plate. Figure 2 (upper panel) shows that biotinylated BL/6 anti-C3H IgG binds to C3H anti-BL/6 IgG and to C3H anti-BL/6 IgG absorbed with BL/6 lymphocytes, but not to normal C3H IgG, nor to normal BL/6 IgG. This is ascribed to the presence of C3H anti-anti-C3H antibodies in the C3H anti-BL/6 IgG and in the C3H anti-BL/6 IgG absorbed with BL/6 lymphocytes. Absorption with BL/6 lymphocytes removes antigen-specific (anti‑BL/6) antibodies without removing the anti-idiotypic antibodies. Additional controls are BL/6 anti-C3H and BL/6 anti-C3H absorbed with C3H lymphocytes. Conversely, the lower panel in Figure 2 shows biotinylated C3H anti-BL/6 IgG binds to BL/6 anti-C3H IgG and to BL/6 anti-C3H IgG absorbed with C3H lymphocytes, but not to normal BL/6 IgG, nor to normal C3H IgG. Additional controls used were C3H anti-BL/6 and C3H anti-BL/6 absorbed with BL/6 lymphocytes.

Figure 3 highlights a symmetrical immune network model that shows how the V regions of helper T cells, suppressor T cells, IgG secreting B cells, and IgM secreting B cells may be related to each other [6]. This figure purports to show the relationships between the various populations independently of any perturbation by an antigen. T suppressor (Ts) 1 cells express CD4 and are postulated to be regulator T (Treg) cells. Ts2 cells are at the center of the network. They express CD8 and include classic suppressor T cells. When there is a significant change in B cells secreting IgG specific for an antigen such as foreign MHC class I as the result of skin grafting, this impacts on all the populations shown including Ts2 and IgG secreting cells (B2) that as a class are believed to be anti-idiotypic to Ts1 and Ts3 lymphocytes, and in this respect are anti-anti-self.

When there is a significant change in the B2 lymphocyte population following exposure to an antigen, with the production of antigen-specific antibodies, there are plausibly changes in the other populations due to the couplings shown in Figure 3. Our findings are most simply explained in terms of the hypothesis that new immune responses with the production of IgG generally include the production of both new antigen-specific antibodies and new anti-idiotypic antibodies (anti-anti-self).

### Antigen-Specific and Anti-Idiotypic Antibodies in Mice Immunized With Conventional Ag

In our first tests of the explanatory value of the symmetrical immune network model, we measured the production of antigen-specific and anti-idiotypic antibodies in BL/6 and C3H mice immunized with tetanus toxoid (Td) in the adjuvant monophosphoryl lipid A adjuvant (MPLA). Six BL/6 mice and 6 C3H mice were immunized on day 0 and day 14 and bled on day 21. Normal IgG, IgG purified from the immunized mice, or IgG from the immunized mice that had been absorbed with Td were coated on ELISA plates at the dilutions shown in Figure 3. Data in the panels of this Figure are pooled from 2 similar studies (total of 10 mice/group). Figure 3 shows that biotinylated BL/6 anti-C3H IgG (prepared from a pool of 15 mice immunized as described earlier by repeated skin grafts) binds to C3H anti-Td IgG and to C3H anti-Td IgG absorbed with Td but not to normal C3H IgG, nor to normal BL/6 IgG. This is ascribed to the presence of C3H anti-anti-C3H antibodies in the C3H anti-Td IgG and in the C3H anti-Td IgG absorbed with Td. Additional controls are C3H anti-Td and BL/6 anti-Td absorbed with Td. Figure 3 also shows that biotinylated C3H anti-BL/6 IgG (again pooled from 15 grafted donors) binds to BL/6 anti-Td IgG and to BL/6 anti-Td IgG absorbed with Td but not to normal BL/6 IgG, nor to normal C3H IgG. Additional controls are C3H anti-Td and C3H anti-Td absorbed with Td. This confirms that the IgG immune response of C3H and of BL/6 mice to Td includes the production of C3H anti-anti-C3H and BL/6 anti-anti-BL/6 antibodies, respectively.

### Induction of Cytokines and Regulatory T Cells (Tregs) or Suppressor T Cells by Anti-Idiotypic Antibody

Further analysis of the functional properties of anti-idiotypic IgG is shown in Figure 4 where we indicate that anti-idiotypic antibodies can stimulate the production of cytokines and induce regulatory T cells. Figure 4 shows cytokine levels induced in cultures of C3H splenocytes (pooled from 5 naïve mice) at 40 h with no stimulation, and with stimulation by 20μg/ml of either normal BL/6 IgG or BL/6 anti-anti-BL/6 IgG (left hand of panel), whereas the right hand side of the same panel shows the cytokine levels induced in cultures of BL/6 splenocytes with no stimulation, and with stimulation by either normal C3H IgG or C3H anti-anti-C3H IgG. The cytokine levels were measured in duplicate by ELISA. All data are pooled from 2 similar studies. Stimulation by the anti-anti-self antibodies induced secretion of the inflammatory cytokines IFNγ, TNFα, and IL-6, with no measurable secretion of the anti-inflammatory cytokine IL-10. Interestingly, IL-4 and TGFβ production were also stimulated. The former is often used as a marker of Th2 stimulation and augments B-cell Ig production, whereas TGFβ is a key cytokine implicated in development and/or expansion of Tregs (Ts1 cells).

In many cases, when T cells from a mouse that has been primed with an antigen are combined with naïve cells in a recipient mouse, the mouse is specifically suppressed for responses to that antigen. This is the classic suppressor T cell phenomenon [25,26], in which the suppressor T cells express the CD8 marker and should not be confused with the CD4+, CD25+ Treg cells described elsewhere [27]. The CD8 suppressor T cell phenomenon can be understood in terms of coselection of antigen‑specific cells and anti-idiotypic T cells. In the context of the model in Figure 3, Treg cells are interpreted as being Ts1 cells (CD4) and classic suppressor T cells are Ts2 cells (CD8).

From the study shown in the left panels of Figure 4, the cells stimulated to produce cytokines were harvested following incubation with anti-idiotypic antibody and tested for their ability to suppress the induction of cytotoxic T cells in a subsequent culture. The results are shown in Figure 4 (right hand panel), again pooled from the 2 independent studies. Lymphocytes pooled from triplicate C3H cultures that had received BL/6 anti-anti-BL/6 IgG antibodies suppressed the cytotoxic T lymphocytes (CTL) response of C3H lymphocytes to BL/6 lymphocytes, and lymphocytes pooled from triplicate BL/6 cultures that had been treated with C3H anti-anti-C3H suppressed the CTL response of BL/6 lymphocytes to C3H. Antigen-specific suppression is a known property of both CD8 suppressor T cells and of so-called inducible CD4+ Tregs (iTregs [CD4^+^])—importantly, we acknowledge that the phenotype of the Tregs measured here remains to be determined. Taken in combination, the anti-anti-self-Ig mediated induction of cytokine production and Tregs is taken to reflect a role for such antibodies in activating a “network” of immunoregulation through complementary cell surface receptors.

### Synergy Between Antigen-Specific Plus Anti-Idiotypic Antibodies in Inducing Graft Tolerance

The second symmetry relationships shown in Figure 3 is also postulated to provide a mechanism of inducing transplantation tolerance using coselection. A vertebrate A can be treated with a combination of A anti-B antibodies (antigen-specific) and B anti-anti-B antibodies (anti-idiotypic) to induce a state in which there is transplantation tolerance in A that is specific for B. The envisaged mechanism is shown in Figure 5. The anti-B antibodies stimulate anti-anti-B T cells and the anti-anti-B antibodies stimulate anti-B T cells. There is coselection of the anti-anti-B T cells and the anti-B T cells, taking the immune system of A to a state in which there are elevated levels of these 2 T cell populations. In the case of this tissue transplant model, this state is predicted to be one in which A is specifically unresponsive to B. A more detailed methodology for producing complementary pairs of antibodies for use in protocols designed to vaccinate animals to augment immunoregulatory circuits is shown in the lower left hand panel in Figure 5.

In an experiment designed to test the validity of this concept, A is represented by C3H mice and B by BL/6 mice. Groups of 20 C3H mice were infused with a combination of C3H anti-BL/6 antibodies and BL/6 anti-anti-BL/6 antibodies. We then sacrificed mice and tested for induction of anti-BL/6 CTL following stimulation with irradiated BL/6 splenocytes in vitro. The C3H anti-BL/6 IgG antibodies were contained in serum obtained from a pool of 15 C3H mice that had undergone 2 rounds of skin grafting with BL/6 skin. The BL/6 anti-anti-BL/6 antibodies were obtained from a pool of 15 BL/6 mice that had undergone 2 rounds of skin grafting with C3H skin. The IgG from the latter mice was absorbed with C3H spleen cells and thymocytes until all anti-C3H antibodies had been removed (see Methods).

Spleen cells from the treated C3H mice were tested for tolerance by exploring the attenuation of the induction of BL/6‑specific CTL in response to stimulation by irradiated BL/6 spleen cells as the assay system. There were 2 groups of 20 C3H mice each, with group 1 being infused with anti-BL/6 plus BL/6 anti-anti-BL/6 antibodies given IV, whereas in group 2, the antibodies were given IP.

All C3H mice received weekly infusions of 1µg of BL/6 anti-anti-BL/6 antibodies plus 1µg C3H anti-BL/6 antibodies beginning on day 0. Mixed lymphocyte cultures using cells from 4 mice per group were set up at days 14, 21, 28, 35, and 42, with BL/6 spleen cells as the stimulators. The results are shown in the serial panels to the right in Figure 5. The negative control is the result for stimulation of cells from untreated C3H mice by BL/6 spleen cells, and the positive control is the result for induction of a BALB/c-specific CTL in response to stimulation by BALB/c lymphocytes. A highly significant reduction in the BL/6-specific CTL response was observed for the cultures set up after 4, 5, and 6 infusions of the antibodies. There was no significant difference between intraperitoneal and intravenous administration of the antibodies. Similar data were observed in a repeat study of the same type.

We conclude that stimulation of an immune system by antigen-specific (for example anti-B) plus complementary anti-idiotypic antibodies (anti-anti-B) can induce a new stable steady state in which the immune response to B is specifically suppressed.

### Therapeutic Effect of Anti-Anti-Self Plus Complementary Antiforeign Antibodies in an Inflammatory Bowel Disease (IBD) Model

IBD is a category of autoimmune diseases that includes colitis and Crohn disease. BL/6 mice fed DSS develop IBD [20]. We tested the efficacy of prevention of IBD by antigen-specific plus anti-idiotypic antibodies in an experiment with 3 groups of 8 BL/6 mice. The first group was given normal drinking water and a normal diet, the second was given a diet that included DSS and high fat, and the third was given DSS and high fat plus 2 infusions of antigen-specific (BL/6 anti-C3H), plus anti-idiotypic (C3H anti-anti-C3H) antibodies for 2 weeks at days −14 and −7 before commencement of the diet of the DSS plus high fat diet at day 0. The mice were successfully treated by the combination of antibodies as measured in 3 assays, namely reduction of weight loss reduction in the change of colon length, and inhibition of the production of mRNA for 7 of 9 inflammatory cytokines (see respective panels in Figure 6). We attribute this therapeutic effect to changing the phenotype of the treated mice to have similarity to that of C3H mice, while not losing tolerance to the BL/6 phenotype, meaning self‑tolerance to BL/6 is not lost. The treated BL/6 mice have the benefit of tolerance to both BL/6 and C3H, while the ability to respond to other antigens is fully retained. In a control experiment (not shown), the mice were treated with only C3H anti-anti-C3H antibodies. The suppression of inflammation was not observed. We conclude that treatment with antigen-specific plus anti-idiotypic antibodies as described here is effective in the prevention of inflammatory bowel disease.

### Therapeutic Effect of Antigen-Specific Plus Anti-Idiotypic Antibodies in an Allergy Model

To assess whether this same strategy could be used to attenuate allergic diseases, we performed a study in 8 per group BALB/c mice immunized with OVA in alum and subsequently exposed to egg white solution in the drinking water—see Methods and [21]. After 7 d following a first immunization with OVA, subgroups of mice were given infusions of antigen-specific (BL/6 anti-C3H) or anti-idiotypic (C3H anti-anti-C3H) Ig alone, or in combination, along with egg white solution. A total of 5 immunizations was given at weekly intervals. Following 7 d after the last infusion, all mice were returned to normal drinking water, boosted with a final dose of OVA in alum, and sacrificed 7d later for measurement of serum IgE, and induction of IL-4 by OVA-stimulated splenocytes in vitro (see Figure 7).

It is apparent from these data that indeed, combination Ig treatment does indeed markedly attenuate IgE production. In accord with this finding, such treated mice showed a marked reduction in IL-4 release following OVA-restimulation.

### Therapeutic Effect of Antigen-Specific Plus Anti-Idiotypic Antibodies in a Cancer Model

EMT6 is a transplantable breast cancer tumor [22]. A total of 6 mice per group received 5x10^5^ EMT6 cells 14d after 2 intravenous injections of C3H anti-BL/6 and/or BL/6 anti-anti-BL/6 IgG (10μg/mouse). Injections were continued at 7 d intervals thereafter until the final sacrifice. After 14 d from receiving the EMT6 injection, mice were anesthetized and tumors resected and weighed. Mice were returned to their cages for a further 16 d when all were sacrificed and lung nodules evaluated. The results for tumor size and the number of metastases in the lungs are shown in Figure 8 (upper left hand panel). The tumor size and the number of metastases was significantly less in the mice that received C3H anti-BL/6 plus BL/6 anti-anti-BL/6 IgG.

Cytokine data for this experiment is shown in the right hand panels in this Figure. Lymph nodes draining the tumor were harvested at sacrifice, and 1x10^6^ cells were cultured in duplicate in 1ml medium with either 1x10^6^ irradiated (2500Rads) BL/6 splenocytes or 1x10^5^ irradiated EMT6 tumor cells. Supernatants were harvested at 48 h and assayed in commercial ELISAs (BioLegend) for the cytokines shown. The results for stimulation of BL/6 spleen cells (H‑2^b^) and of EMT6 (H-2^d^) were the same. IL-1β and IL-6 were significantly down‑regulated, whereas TGFβ was significantly up‑regulated. IL‑1β and IL-6 are proinflammatory cytokines, whereas TGFβ regulates inflammation. There was no change in the level of TNFα. Hence, for three of the four cytokines, there was a significant therapeutic effect resulting from the infusions of the antigen-specific plus anti-idiotypic antibodies

## Discussion

### Principal Findings

The data described above have focused on documenting evidence that infusion of a combination of A anti-B and anti-anti-A antibodies can lead to development of an altered immune state in recipient animals in which attenuation of immunity to an antigen simultaneously administered can be achieved. We have investigated evidence for this hypothesis in models of nominal antigen immunization, of graft specific allo-tolerance, of IBD, of OVA-induced IgE production, and finally of breast cancer metastasis. In all scenarios, we observed a significantly altered immune state in antigen-challenged individuals.

A great deal of research on anti-idiotypic antibodies has been based on the idea that anti-idiotypic antibodies can mimic the antigen. There was optimism that such antibodies could be effective as vaccines, but this line of research has not led to the commercialization of any vaccines over the last 40 years. The symmetrical immune network theory has been developed over the same 40 years and is able to account for many aspects of the adaptive immune system [4-7]. An important aspect of the theory is that it is based on the well-established; nonetheless, controversial presumed existence of specific T cell factors [8-17]. As a first important step in documenting the validity of the underlying concept of symmetrical immune network theory, we confirmed in this paper (see Figure1b) that such factors do indeed exist, as initially postulated by Takemori and Tada [10].

We find that normal IgG immune responses in a vertebrate against an antigen consist of 2 components, namely antigen-specific, and second symmetry anti-idiotypic antibodies. The second symmetry anti-idiotypic antibodies in an immune response of a vertebrate A to skin grafts from a vertebrate B bind to antigen-specific antibodies present in a B anti-A immune response. The IgG of a vertebrate A immune to the protein antigen tetanus toxoid likewise includes anti-idiotypic antibodies (A anti-anti-A) that are specific for antigen-specific antibodies (B anti-A) present in a B anti-A immune response—again data in Figures 2 and 3 support these concepts.

A combination of antigen-specific plus second symmetry anti-idiotypic antibodies is effective in inducing allograft tolerance. The proposed mechanism for this is that there is coselection of antigen-specific and complementary anti-idiotypic T cells. Data in a skin allograft model, shown in Figures 4 and 5, are in accord with these hypotheses also. Induction of anti-idiotypic regulatory cells is postulated to underlie the mechanism whereby this same approach could be used to down-regulate inflammation in a murine model of IBD (Figure 6) as measured in each of 3 assays, namely by monitoring weight loss (Figure 6), colon length (Figure 6), or mRNA expression of chemokines or cytokines (Figure 6). We also report that the same antibody combinations can be used successfully to attenuate allergic responses (IgE production after immunization of mice with OVA in alum, and production of IL-4 cytokines in these same mice—see Figure 7), and of inhibiting tumor growth and preventing metastases in the EMT6 mouse breast cancer model (Figure 8). In this latter case, two of three proinflammatory cytokines measured were down-regulated (IL1β, IL-6) and an immunoregulatory cytokine (TGFβ) was up-regulated (Figure 8). These data are consistent with the growing interest in altering immunosurveillance as a tool in regulating cancer metastasis [28]. It is worthwhile noting at this stage that all of the model systems described to test our hypotheses use acute perturbations of the immune system (transplantation, DSS exposure, tumor growth after transplant of tumor cells, and IgE response to acute allergen exposure). To date, we have little information on whether, and for how long, these altered immunregulatory states are maintained in the absence of ongoing antigen exposure, and whether unexpected side-effects (eg, altered autoantibody production) are incurred following antibody infusion, an important issue to answer before application of this therapy in clinical situations. Studies addressing these issues are in progress.

Our experiments indicate that the immune systems of 2 healthy vertebrates called A and B can be combined with the immune system of a third healthy vertebrate C to make C’s immune system resistant to degenerative diseases. A, B, and C may be three strains of mice, and in the case of the application to the immunotherapy to humans, A and B may be mouse strains and C may be a healthy human. Anti-B antibodies may be obtained from immunization of A with B tissue, and anti-anti-B antibodies may be obtained from immunization of B with A tissue. C is then treated with a combination of anti-B and anti‑anti-B antibodies. Note, however, that where the vertebrates used to produce the IgGs (A and B above) are of a different species (mouse) from those (C above) receiving the combined antibodies, we would anticipate a need for “humanizing” the IgGs infused to avoid a confounding human antimouse antibody (HAMA) response [29].

**Figure 9:**
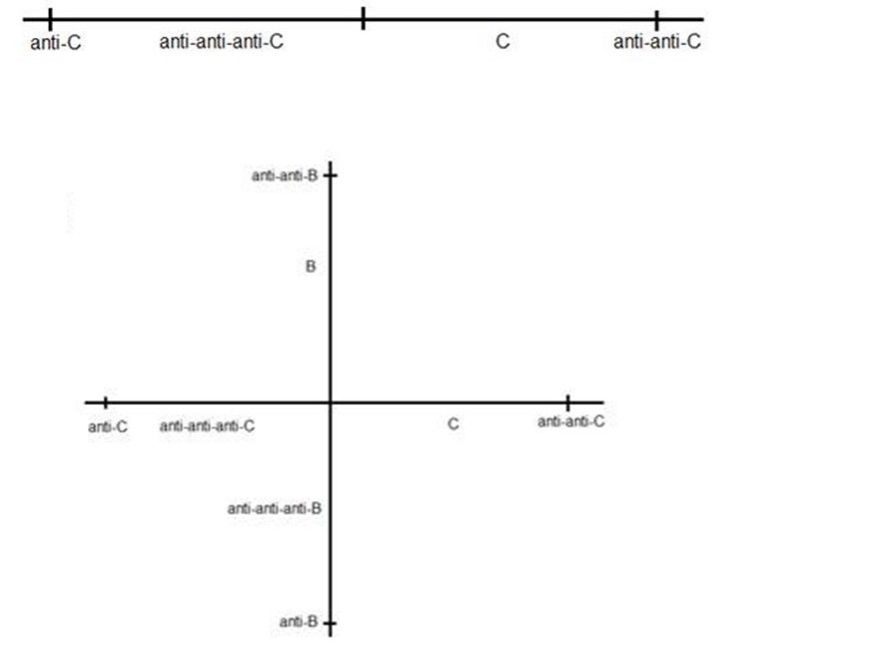
A one-dimensional shape space for the vertebrate C in which complementary shapes map to opposite sides of the origin (upper panel).A two-dimensional shape space results when the vertebrate C is treated with anti-B plus anti-anti-B antibodies. Anti-anti-C and anti-anti-B lymphocytes are co-selected with anti-C and anti-B lymphocytes respectively (lower panel).

Why has evolution not led to normal immune systems being similarly resistant to degenerative diseases? In order to address this question, we would like to first explain that according to the symmetrical immune network theory [6], normal immune systems include self antigens C, lymphocytes that are anti-C and are coselected with anti-anti-C lymphocytes, and lymphocytes that are anti-anti-anti-C and are also coselected with anti-anti-C lymphocytes. The antigens C include especially MHC class II antigens that stimulate the anti-C Th1 lymphocytes and Ts1 lymphocytes. The anti-anti-C lymphocytes include Th2, Ts2, and B2 lymphocytes, whereby B2 lymphocytes are predominantly follicular (FO) B cells or marginal zone (MZ) B cells that secrete IgG following T-dependent activation [30]. The anti‑anti-anti-C lymphocytes include Ts3 and B1 lymphocytes. B1 lymphocytes arise from a developmental pathway different from that of FO B cells and MZ B cells, are primarily activated through T-independent mechanisms, and in mice, populate mainly the peritoneal and pleural cavity where they generate so-called IgM (antibodies produced without infection), which defend against mucosal pathogens [31]. There is therefore a principle “shape space axis” comprising C, anti-C, anti-anti-C, and anti-anti-anti-C. Each of the members in this sequence has shapes that are complementary to the neighboring members in the sequence. We can also depict the shape space axis in a way such that neighboring members are similar to each other and members with complementary shapes are on opposite sides of the diagram. This is shown in Figure 9 (upper panel), in which the axis is defined by anti-C and anti-anti-C. C is complementary to anti-C, so it is on the same side as anti-anti-C, and anti-anti-anti-C is complementary to anti-anti-C, so it is on the same side as anti-C. The system is stabilized by anti-C being stimulated by anti-anti-C and vice versa. At any given time point, there would be a mixture of anti-C and anti-anti-C factors on the A cell surface, and we could expect random fluctuations in which of the two is present to a greater degree. Such fluctuations would result in fluctuations in the number of anti-C and anti-anti-C lymphocytes. There may then be a random walk in one dimension for the relative amounts of anti-C and anti-anti-C lymphocytes. At some stage this random walk could lead to the 2 populations no longer being mutually stabilizing. This idea is supported by the fact that autoimmune mice make anti-anti-C antibodies [20], which could be due to T cells no longer dominating the anti-anti-C region of shape space. In addition, we have found that old BL/6 mice make anti-anti-BL/6 (or “anti-anti-C”) antibodies [21].

When we treat C with anti-B plus anti-anti-B antibodies, we induce a new stable steady state with elevated levels of anti-anti-B and anti-B T cells. This amounts to creating a second shape space axis for the T cells of C that is plausibly orthogonally to the anti‑C or anti‑anti-C shape space axis because neither anti-B nor anti-anti-B has any relation to anti-C or anti-anti-C—see Figure 9 (lower panel). In this case, fluctuations in all of anti-C, anti‑anti-C, anti-B, and anti-anti-B specific T cell factors would mean there is a random walk in two dimensions rather than one dimension. An immune system with a single shape space axis as shown in Figure 9 may be intrinsically less stable that a system with a second shape space axis. In the case of the former, the steady state involves primarily just anti-C and anti‑anti-C T cell factors on the A cell surface, whereas there are four specificities of specific T cell factors for a system with 2 shape space axes. This difference in the number of shape space dimensions may be the basis for an immune system constructed using three immune systems (A, B, and C), and having 2 shape space axes, being intrinsically stronger than an immune system based on a single shape space axis.

### Conclusions

We conclude that the symmetrical immune network theory leads to a possible preventive immunotherapy that does not involve the production of antibodies and can reasonably be called a new class of vaccine. Combinations of antigen-specific antibodies and second symmetry anti-idiotypic antibodies as described here comprise an immunotherapy for the prevention of IBD, for attenuation of allergic responses, and the prevention of breast cancer. The same therapy may prove to be effective in preventing also other autoimmune diseases and cancers, and as such, likely represents an important new addition to our clinical armamentarium.

## Abbreviations

BGG: bovine gamma globulin
CTL: cytotoxic T lymphocytes
DNP: dinitrophenol
DSS: dextran sulfate sodium
ELISA: enzyme-linked immunosorbent assay
GAPDH: glyceraldehyde 3-phosphate dehydrogenase
HAMA: human antimouse antibody
HPRT: hypoxanthine-guanine phosphoribosyltransferase
HRP: horseradish peroxidase
IBD: inflammatory bowel disease
KLH: keyhole limpet hemocyanin
MPLA: monophosphoryl lipid A adjuvant
OVA: ovalbumin
PBS: phosphate buffered saline
RNA: ribonucleic acid
RT-PCR: reverse transcription polymerase chain reaction
Ts: T suppressor cells
Treg: regulatory T cells
Td: tetanus toxoid
UHN: University Health Network
